# Prospective analysis of circulating metabolites and breast cancer in EPIC

**DOI:** 10.1186/s12916-019-1408-4

**Published:** 2019-09-24

**Authors:** Mathilde His, Vivian Viallon, Laure Dossus, Audrey Gicquiau, David Achaintre, Augustin Scalbert, Pietro Ferrari, Isabelle Romieu, N. Charlotte Onland-Moret, Elisabete Weiderpass, Christina C. Dahm, Kim Overvad, Anja Olsen, Anne Tjønneland, Agnès Fournier, Joseph A. Rothwell, Gianluca Severi, Tilman Kühn, Renée T. Fortner, Heiner Boeing, Antonia Trichopoulou, Anna Karakatsani, Georgia Martimianaki, Giovanna Masala, Sabina Sieri, Rosario Tumino, Paolo Vineis, Salvatore Panico, Carla H. van Gils, Therese H. Nøst, Torkjel M. Sandanger, Guri Skeie, J. Ramón Quirós, Antonio Agudo, Maria-Jose Sánchez, Pilar Amiano, José María Huerta, Eva Ardanaz, Julie A. Schmidt, Ruth C. Travis, Elio Riboli, Konstantinos K. Tsilidis, Sofia Christakoudi, Marc J. Gunter, Sabina Rinaldi

**Affiliations:** 1 International Agency for Research on Cancer, 150 cours Albert Thomas, 69372 Lyon CEDEX 08, France; 2 Centre for Research on Population Health, National Institute of Public Health, Cuernavaca, Mexico; 3 Julius Center for Health Sciences and Primary Care, University Medical Center Utrecht, Utrecht University, Utrecht, the Netherlands; 4 Department of Public Health, Aarhus University, Aarhus, Denmark; 5 Department of Cardiology, Aalborg University Hospital, Aalborg, Denmark; 6 Danish Cancer Society Research Center, Copenhagen, Denmark; 7 University of Copenhagen, Copenhagen, Denmark; 8 CESP, Université Paris-Sud, UVSQ, INSERM, Université Paris-Saclay, Villejuif, France; 9 Gustave Roussy, Villejuif, France; 10 Division of Cancer Epidemiology, German Cancer Research Center (DKFZ), Heidelberg, Germany; 11 Department of Epidemiology, German Institute of Human Nutrition Potsdam-Rehbruecke (DIfE), Arthur-Scheunert-Allee 114-116, 14558 Nuthetal, Germany; 12 Hellenic Health Foundation, Athens, Greece; 13 Pulmonary Medicine Department, School of Medicine, National and Kapodistrian University of Athens, “ATTIKON” University Hospital, Haidari, Greece; 14 Cancer Risk Factors and Life-Style Epidemiology Unit, Institute for Cancer Research, Prevention and Clinical Network – ISPRO, Florence, Italy; 15 Epidemiology and Prevention Unit, Fondazione IRCCS Istituto Nazionale dei Tumori di Milano, Milan, Italy; 16 Cancer Registry and Histopathology Department, “M.P.Arezzo”Hospital, ASP Ragusa, Ragusa, Italy; 17 Italian Institute for Genomic Medicine (IIGM), 10126 Turin, Italy; 18 MRC-PHE Centre for Environment and Health, School of Public Health, Imperial College London, London, UK; 19 Dipartimento di medicina clinica e chirurgia, Federico II University, Naples, Italy; 20 Department of Community Medicine, UiT the Arctic University of Norway, Tromso, Norway; 21 Nutritional Epidemiology Group, School of Food Science and Nutrition, University of Leeds, Leeds, UK; 22 Public Health Directorate, Asturias, Spain; 23 Unit of Nutrition and Cancer, Cancer Epidemiology Research Program, Catalan Institute of Oncology-IDIBELL, L’Hospitalet de Llobregat, Barcelona, Spain; 24 Escuela Andaluza de Salud Pública, Instituto de Investigación Biosanitaria ibs.GRANADA, Universidad de Granada, Granada, Spain; 25 CIBER Epidemiology and Public Health CIBERESP, Madrid, Spain; 26 Public Health Division of Gipuzkoa, BioDonostia Research Institute, San Sebastian, Spain; 27 Department of Epidemiology, Murcia Regional Health Council, IMIB-Arrixaca, Murcia, Spain; 28 Navarra Public Health Institute, Pamplona, Spain; 29 IdiSNA, Navarra Institute for Health Research, Pamplona, Spain; 30 Cancer Epidemiology Unit, Nuffield Department of Population Health, University of Oxford, Oxford, UK; 31 Department of Epidemiology and Biostatistics, Imperial College London, St Mary’s Campus, Norfolk Place, London, W2 1PG UK; 32 Department of Hygiene and Epidemiology, University of Ioannina School of Medicine, Ioannina, Greece; 33 MRC Centre for Transplantation, King’s College London, Great Maze Pond, London, SE1 9RT UK

**Keywords:** Breast cancer, Metabolomics, Prospective study

## Abstract

**Background:**

Metabolomics is a promising molecular tool to identify novel etiologic pathways leading to cancer. Using a targeted approach, we prospectively investigated the associations between metabolite concentrations in plasma and breast cancer risk.

**Methods:**

A nested case-control study was established within the European Prospective Investigation into Cancer cohort, which included 1624 first primary incident invasive breast cancer cases (with known estrogen and progesterone receptor and HER2 status) and 1624 matched controls. Metabolites (*n* = 127, acylcarnitines, amino acids, biogenic amines, glycerophospholipids, hexose, sphingolipids) were measured by mass spectrometry in pre-diagnostic plasma samples and tested for associations with breast cancer incidence using multivariable conditional logistic regression.

**Results:**

Among women not using hormones at baseline (*n* = 2248), and after control for multiple tests, concentrations of arginine (odds ratio [OR] per SD = 0.79, 95% confidence interval [CI] = 0.70–0.90), asparagine (OR = 0.83 (0.74–0.92)), and phosphatidylcholines (PCs) ae C36:3 (OR = 0.83 (0.76–0.90)), aa C36:3 (OR = 0.84 (0.77–0.93)), ae C34:2 (OR = 0.85 (0.78–0.94)), ae C36:2 (OR = 0.85 (0.78–0.88)), and ae C38:2 (OR = 0.84 (0.76–0.93)) were inversely associated with breast cancer risk, while the acylcarnitine C2 (OR = 1.23 (1.11–1.35)) was positively associated with disease risk. In the overall population, C2 (OR = 1.15 (1.06–1.24)) and PC ae C36:3 (OR = 0.88 (0.82–0.95)) were associated with risk of breast cancer, and these relationships did not differ by breast cancer subtype, age at diagnosis, fasting status, menopausal status, or adiposity.

**Conclusions:**

These findings point to potentially novel pathways and biomarkers of breast cancer development. Results warrant replication in other epidemiological studies.

**Electronic supplementary material:**

The online version of this article (10.1186/s12916-019-1408-4) contains supplementary material, which is available to authorized users.

## Background

Breast cancer is the most common cancer among women worldwide [1]. Known modifiable hormonal and lifestyle risk factors, however, are estimated to be responsible for only around 30% of breast cancers in high-income countries [2–8], so a better understanding of the etiology of the disease and of the biological mechanisms is needed.

The metabolome reflects endogenous processes and environmental and lifestyle factors [9–13]. Metabolomics can detect subtle differences in metabolism; therefore, it is a promising tool to identify new etiological pathways. Previous prospective studies of breast cancer which have employed metabolomics have used both targeted (analyses of a pre-defined panel of metabolites) [14] or untargeted (where as many metabolites as possible are measured and then characterized [15]) approaches [16–18]. In previous studies, lysophosphatidylcholine a C18:0 [14], various lipids, acetone, and glycerol-derived compounds [16], 16a-hydroxy-DHEA-3-sulfate, 3-methylglutarylcarnitine [17], and caprate (10:0), were associated with breast cancer development [18]. The number of cases included in these studies was, however, limited (from 200 to 621) and heterogeneity by subtype was investigated in only one study [18].

In the current study, we employed a targeted metabolomics approach to prospectively investigate the associations between 127 metabolites measured by mass spectrometry in pre-diagnostic plasma samples and risk of breast cancer, overall, and by breast cancer subtype, accounting for established breast cancer risk factors.

## Methods

### Study population, blood collection, and follow-up

EPIC is an ongoing multi-center cohort study including approximately 520,000 participants recruited between 1992 and 2000 from ten European countries [19]. Female participants (*n* = 367,903) were aged 35–75 years old at inclusion. At recruitment, detailed information was collected on dietary, lifestyle, reproductive, medical, and anthropometric data [19]. Around 246,000 women from all countries provided a baseline blood sample. Blood was collected according to a standardized protocol in France, Germany, Greece, Italy, the Netherlands, Norway, Spain, and the UK [19]. Serum (except in Norway), plasma, erythrocytes, and buffy coat aliquots were stored in liquid nitrogen (− 196 °C) in a centralized biobank at IARC. In Denmark, blood fractions were stored locally in the vapor phase of liquid nitrogen containers (− 150 °C), and in Sweden, they were stored locally at − 80 °C in standard freezers.

Incident cancer cases were identified through record linkage with cancer registries in most countries and through health insurance records, cancer and pathology registries, and active follow-up of study subjects in France, Germany, and Greece. For each EPIC center, closure dates of the study period were defined as the latest dates of complete follow-up for both cancer incidence and vital status (dates varied between centers, from June 2008 to December 2012).

All participants provided written informed consent to participate in the EPIC study. This study was approved by the ethics committee of the International Agency for Research on Cancer (IARC) and all centers.

### Selection of cases and controls

Subjects were selected among participants who were cancer-free (other than non-melanoma skin cancer) and had donated blood at recruitment into the cohort. Cancers were coded according to the Third Edition of the International Classification of Diseases for Oncology (code C50). Women diagnosed with first primary invasive breast cancer at least 2 years after blood collection and before December 2012, for whom estrogen receptor (ER), progesterone receptor (PR), and human epidermal growth factor receptor 2 (HER2) statuses of the tumors were available, were selected as cases for the current study.

For each breast cancer case, one control was chosen at random among appropriate risk sets comprising all female cohort members who were alive and without cancer diagnosis (except non-melanoma skin cancer) at the time of diagnosis of the index case. Using incidence density sampling, controls were matched to cases on center of recruitment, age (± 6 months), menopausal status (premenopausal, perimenopausal, postmenopausal, surgically postmenopausal [20]), phase of the menstrual cycle [20], use of exogenous hormone at blood collection, time of the day (± 1 h), and fasting status at blood collection (non-fasting (< 3 h since last meal), in between (3–6 h), fasting (> 6 h), unknown).

Initially, 1626 cases and 1626 controls were eligible for the study, but after the exclusion of pregnant women at blood collection, a final population of 1624 cases and 1624 controls were included in the analysis.

### Laboratory measurements

All plasma samples were assayed in the Biomarkers laboratory at IARC, using the Absolute*IDQ* p180 platform (Biocrates Life Sciences AG, Innsbruck, Austria) and following the procedure recommended by the vendor. A QTRAP5500 mass spectrometer (AB Sciex, Framingham, MA, USA) was used to measure 147 metabolites (19 acylcarnitines, 21 amino acids, 13 biogenic amines, 79 glycerophospholipids, 14 sphingolipids and hexoses). Samples from matched case-control sets were assayed in the same analytical batch. Laboratory personnel were blinded to case-control status of the samples.

### Selection of metabolites

Metabolites were analyzed in samples from 3247 distinct subjects (one subject included in 2 pairs). Completeness of measures and coefficients of variation (median = 5.3%, interquartile range = 1.4%) are shown in Additional file 1: Table S1**.** Values lower than the lower limit of quantification (LLOQ), or higher than the upper limit of quantification (ULOQ), as well as lower than batch-specific limit of detection (LOD) (for compounds measured with a semi-quantitative method: acylcarnitines, glycerophospholipids, sphingolipids), were considered out of the measurable range. Metabolites were excluded from the statistical analyses if more than 20% of observations were outside the measurable range (*n* = 20). A total of 127 metabolites (8 acylcarnitines, 20 amino acids, 6 biogenic amines, 78 glycerophospholipids, 14 sphingolipids and hexoses) were finally retained for statistical analyses. Of these 127 metabolites, 113 had all values included in the measurable range. For the remaining 14 metabolites, values outside the quantifiable range (all lower than LLOQ or LOD) were imputed with half the LLOQ or half the batch-specific LOD, respectively.

### Statistical analysis

Characteristics of cases and controls were described using mean and standard deviation (SD) or frequency. Geometric means were used to describe non log-transformed metabolite concentrations among cases and controls. Log-transformed metabolite concentrations were used in all other analyses. Partial Pearson’s correlations between metabolites, adjusted for age at blood collection, were estimated among controls.

We used conditional logistic regression to estimate the risk of breast cancer per standard deviation (SD) increase in metabolite concentration. The analysis was conditioned on the matching variables. Likelihood ratio tests were performed to compare linear models with cubic polynomial models in order to assess departure from linearity. Multiple testing was addressed by controlling for family-wise error rate at *α* = 0.05 by permutation-based stepdown *minP* adjustment of *P* values, as this method better accounts for the dependence of the tests [21, 22]. For comparison with previous studies, we also adjusted the raw *P* values using Bonferroni correction (*P* < 0.05/127) and controlling for the false discovery rate (FDR) at *α* = 0.05 [23]. All statistical tests were two-sided.

Metabolites showing a statistically significant association with risk of breast cancer after correcting for multiple testing were categorized into quintiles based on the distribution of the concentrations among controls, and odds ratios (OR) for risk of breast cancer were estimated in each category. For tests of linear trend, participants were assigned the median value in each quintile and we modeled the corresponding variable as a continuous term. To identify potential confounders, models of the metabolites of interest (continuous and quintiles) were adjusted separately for each potential confounder and estimates obtained were compared with estimates from models with matching variables only. Only variables that changed parameter estimates by more than 10% were retained in the multivariable model. Variables tested were as follows: age at first menstrual period (continuous), number of full-term pregnancies (0/1/2/≥ 3), age at first full-term pregnancy (never pregnant/quartiles), breastfeeding (ever/never/never pregnant/missing; duration in quintiles), ever use of oral contraceptive (yes/no), ever use of MHT (yes/no/missing), smoking status (never/former/current), level of physical activity (Cambridge index [24]: inactive/moderately inactive/moderately active/active), alcohol consumption (nondrinkers/> 0–3/3–12/12–24 g/day), education level (no schooling or primary/technical, professional or secondary/longer education), energy intake (continuous, quintiles), height (continuous, quintiles), sitting height (missing/quartiles), weight (continuous, quintiles), body mass index (continuous, quintiles), waist circumference (continuous, quintiles), hip circumference (continuous, quintiles), and hypertension (yes/no). For these variables, missing values were assigned the median (continuous variables) or mode (categorical variables) if they represented less than 5% of the population, or were otherwise classified in a “missing” category (breastfeeding, ever use of MHT, sitting height). Only waist circumference (continuous), hip circumference (continuous), and weight (continuous) were included in the final models. Given the correlations between these variables (> 0.77), these variables were included separately in three different models.

For those metabolites showing a significant association with breast cancer risk after controlling for multiple testing, heterogeneity was investigated by menopausal status at blood collection, use of exogenous hormones at blood collection, fasting status at blood collection, age at diagnosis (age 50 or older/younger than age 50), breast cancer subtype (ER+PR+/−HER2+, ER+PR+/−HER2−, ER−PR−HER2+, ER−PR−HER2−), time between blood collection diagnosis (2–8.6 years/more than 8.6 years), and at recruitment waist circumference (WC) (< 80 cm/≥80 cm), BMI (< 25 kg/m^2^/≥25 kg/m^2^), and country, by introducing interaction terms in the models. Subgroup analyses were conducted on the raw models. For WC, unconditional logistic regression adjusted for each matching factor was used. *P* values were not corrected for multiple tests since heterogeneity was investigated only for metabolites showing statistically significant associations with risk overall, after correction for multiple testing.

A sensitivity analysis of all 127 metabolites was performed on hormone non-users (1124 cases and 1124 controls) and by cancer subtype.

Analyses were conducted using SAS software for Windows (version 9.4, Copyright© 2017, SAS Institute Inc.) and R software (packages Epi and NPC) [25, 26].

## Results

Cases were diagnosed on average 8.3 years after blood collection, at a mean age of 60.8 years. The majority of tumors were ER-positive (80.7%), PR-positive (68.2%), and HER2-negative (78.2%) (Table 1). Mean concentrations of metabolites by case/control status are shown in Additional file 1: Table S2.

**Table 1:** Main characteristics of the study population

Variables	*N*	Controls	Cases
		*N* = 1624	*N* = 1624
		Mean (SD) or *N* (%)	Mean (SD) or *N* (%)
Age at blood collection (years)	3248	52.5 (7.9)	52.5 (8.0)
Length of follow-up from blood collection (years)	3248	–	8.3 (2.8)
Age at diagnosis (years)	1624	–	60.8 (8.3)
ER status	1624		
Negative		–	313 (19.3)
Positive		–	1311 (80.7)
PR status	1624		
Negative		–	516 (31.8)
Positive		–	1108 (68.2)
HER2 status	1624		
Negative		–	1270 (78.2)
Positive		–	354 (21.8)
Age at first menstrual period (years)	3248	13.1 (1.6)	13.0 (1.5)
Number of full-term pregnancies	3248		
0		215 (13.2)	244 (15.0)
1		253 (15.6)	310 (19.1)
2		729 (44.9)	686 (42.2)
≥ 3		427 (26.3)	384 (23.6)
Age at first full-term pregnancy (years)	2789	24.9 (4.3)	25.3 (4.4)
Ever breastfed	3248		
No		194 (11.9)	206 (12.7)
Yes		1116 (68.7)	1080 (66.5)
Never pregnant		215 (13.2)	244 (15.0)
Missing		99 (6.1)	94 (5.8)
Use of exogenous hormones at blood collection	3248		
No		1124 (69.2)	1130 (69.6)
Yes		492 (30.3)	494 (30.4)
Missing		8 (0.5)	0 (0.0)
Menopausal status at blood collection	3248		
Premenopausal		434 (26.7)	434 (26.7)
Postmenopausal		869 (53.5)	872 (53.7)
Perimenopausal		321 (19.8)	318 (19.6)
Fasting status at blood collection (time since last meal)	3248		
< 3 h		737 (45.4)	731 (45.0)
3–6 h		284 (17.5)	285 (17.5)
> 6 h		580 (35.7)	580 (35.7)
Unknown		23 (1.4)	28 (1.7)
Alcohol consumption at recruitment (g/day)	3248	8.9 (12.2)	10.2 (13.5)
Education level	3248		
Primary/no schooling		610 (37.6)	597 (36.8)
Technical/professional/secondary		687 (42.3)	688 (42.4)
Longer education		327 (20.1)	339 (20.9)
Height (cm)	3248	161.4 (6.6)	162.0 (6.6)
Weight (kg)			
Age at diagnosis < 50 years old	382	63.8 (11.3)	63.0 (10.9)
Age at diagnosis ≥ 50 years old	2866	66.7 (10.8)	68.4 (12.2)
Body mass index (kg/m^2^)			
Age at diagnosis < 50 years old	382	24.2 (4.1)	23.9 (4.2)
Age at diagnosis ≥ 50 years old	2866	25.7 (4.1)	26.1 (4.6)
Waist circumference (cm)			
Age at diagnosis < 50 years old	382	76.8 (9.8)	76.4 (10.3)
Age at diagnosis ≥ 50 years old	2866	81.0 (10.4)	82.5 (11.3)
Hip circumference (cm)			
Age at diagnosis < 50 years old	382	99.0 (8.6)	98.3 (8.4)
Age at diagnosis ≥ 50 years old	2866	101.4 (8.0)	102.7 (9.1)

*ER* estrogen receptor, *HER2* human epidermal growth factor receptor 2, *PR* progesterone receptor

Overall, positive, moderate correlations were observed among some of the amino acids, phosphatidylcholines (PCs), lysoPCs, and sphingomyelins (see Additional file 1: Figure S1); the average absolute correlations within each class was 0.36, 0.39, 0.45, and 0.55, respectively (data not tabulated).

### Associations of metabolites with breast cancer risk

**Fig. 1:**
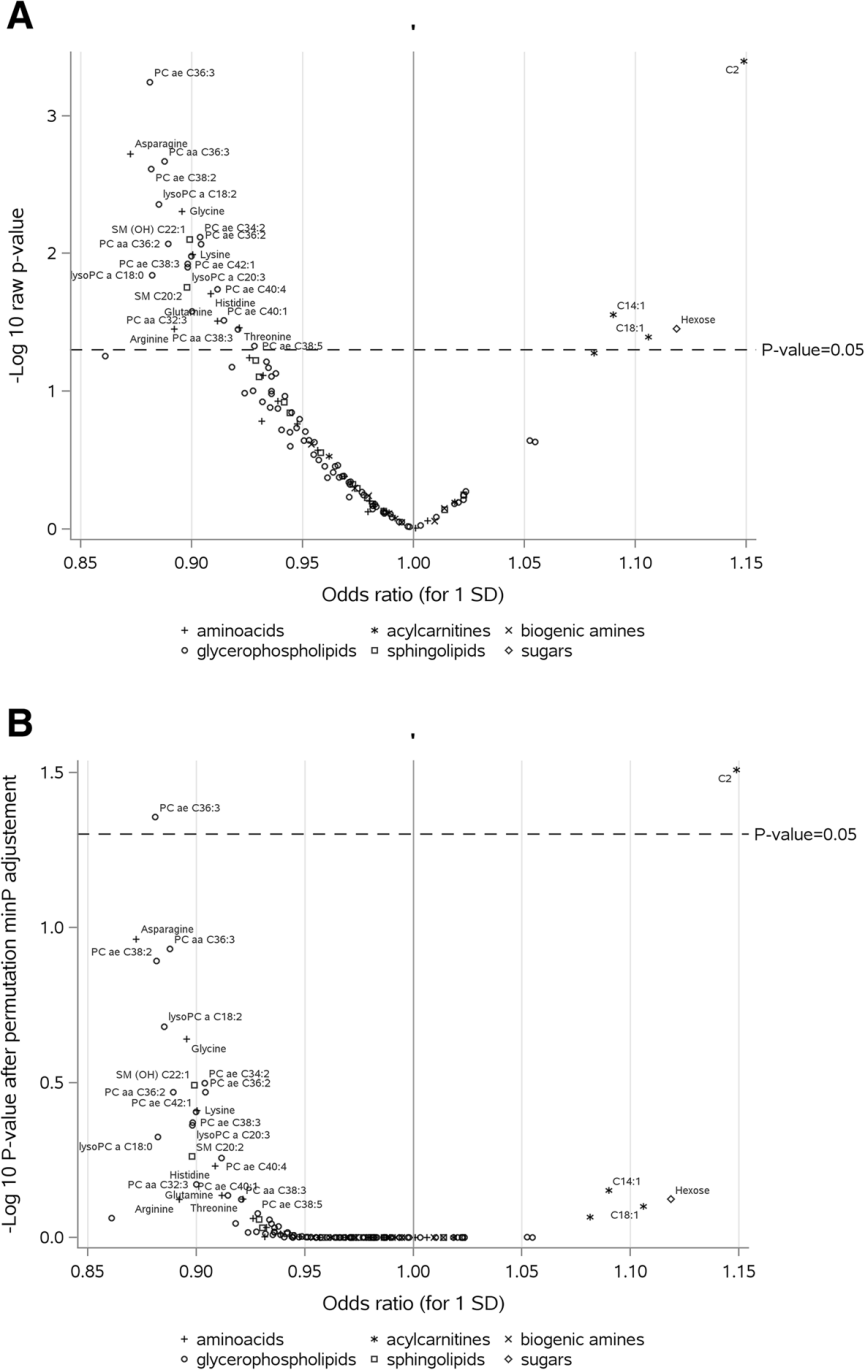
Odds ratios (ORs) for the associations between metabolites and breast cancer. **a** Raw *P* values. **b** Adjusted *P* values. PC: phosphatidylcholine; SM: sphingomyelin. ORs are estimated per standard deviation (SD) increase in log-transformed metabolite concentrations, from logistic regression conditioned on matching variables. **a** Statistical significance based on raw *P* values (significant metabolites above dotted line). **b** Statistical significance based on *P* values adjusted by permutation-based stepdown minP (see “Methods” section for details); adjusted *P* values above 0.05 (dotted line) were considered statistically significant after correction for multiple tests

Prior to correction for multiple testing, 29 metabolites were significantly associated with the risk of breast cancer with a raw *P* value lower than 0.05 (Fig. 1a and Table 2), mainly amino acids, PCs (inversely associated), and acylcarnitines (directly associated). However, after adjusting for multiple testing (Fig. 1b), only C2 (OR for 1 SD increment = 1.15, 95% CI = 1.06–1.24, corrected *P* value = 0.031) and phosphatidylcholine PC ae C36:3 (OR for 1 SD increment = 0.88, 95% CI = 0.82–0.95, corrected *P* value = 0.044) remained significantly associated with risk of breast cancer (Table 2). Adjustment for multiple testing using FDR procedure identified similar significant metabolites, while with Bonferroni correction, only C2 remained associated with risk of breast cancer with a borderline significant *P* value (Bonferroni *P* value = 0.051) (Table 2). Departure from linearity was suggested for glutamate, C0, kynurerine, and SDMA. However, when non-linear models were examined, and after controlling for multiple tests, no non-linear association remained significant (results not shown).

**Table 2:** Associations between metabolites (continuous) and risk of breast cancer, for metabolites with raw *P* values < 0.05

Class	Metabolite	Odds ratio and 95% CI (for 1 SD)^a^	Raw *P* value	Permutation-based minP *P* value^b^	Bonferroni *P* value^c^	False discovery rate *P* value^d^
Amino acids	Arginine	0.89 (0.80–0.99)	0.035	0.753	1.000	0.166
	Asparagine	0.87 (0.80–0.95)	0.002	0.109	0.240	0.062
	Glutamine	0.91 (0.84–0.99)	0.031	0.731	1.000	0.166
	Glycine	0.90 (0.83–0.97)	0.005	0.229	0.629	0.090
	Histidine	0.91 (0.84–0.99)	0.020	0.588	1.000	0.131
	Lysine	0.90 (0.83–0.98)	0.010	0.389	1.000	0.102
	Threonine	0.92 (0.85–0.99)	0.034	0.752	1.000	0.166
Acylcarnitines	C14:1	1.09 (1.01–1.18)	0.028	0.704	1.000	0.166
	C18:1	1.11 (1.00–1.22)	0.040	0.793	1.000	0.183
	*C2*	*1.15 (1.06–1.24)*	*0.0004*	*0.031*	*0.051*	*0.036*
Glycerophospholipids	PC aa C32:3	0.90 (0.82–0.99)	0.026	0.674	1.000	0.166
	PC aa C36:2	0.89 (0.82–0.97)	0.009	0.339	1.000	0.099
	PC aa C36:3	0.89 (0.82–0.96)	0.002	0.117	0.272	0.062
	PC aa C38:3	0.92 (0.85–0.99)	0.035	0.753	1.000	0.166
	PC ae C34:2	0.90 (0.84–0.97)	0.008	0.317	0.966	0.099
	PC ae C36:2	0.90 (0.84–0.98)	0.009	0.339	1.000	0.099
	*PC ae C36:3*	*0.88 (0.82–0.95)*	*0.001*	*0.044*	*0.073*	*0.036*
	PC ae C38:2	0.88 (0.81–0.96)	0.002	0.128	0.310	0.062
	PC ae C38:3	0.90 (0.83–0.98)	0.012	0.425	1.000	0.107
	PC ae C38:5	0.93 (0.86–1.00)	0.047	0.836	1.000	0.205
	PC ae C40:1	0.92 (0.84–0.99)	0.030	0.730	1.000	0.166
	PC ae C40:4	0.91 (0.84–0.98)	0.018	0.553	1.000	0.129
	PC ae C42:1	0.90 (0.83–0.98)	0.010	0.393	1.000	0.102
	lysoPC a C18:0	0.88 (0.80–0.98)	0.014	0.473	1.000	0.115
	lysoPC a C18:2	0.89 (0.81–0.96)	0.004	0.209	0.559	0.090
	lysoPC a C20:3	0.90 (0.83–0.98)	0.013	0.434	1.000	0.107
Sphingolipids	SM C20:2	0.90 (0.82–0.98)	0.018	0.546	1.000	0.129
	SM (OH) C22:1	0.90 (0.83–0.97)	0.008	0.322	1.000	0.099
Sugars	Hexose	1.12 (1.01–1.24)	0.035	0.752	1.000	0.166

*SD* standard deviation, *CI* confidence interval

Italicized text indicates a statistically significant association with breast cancer risk after adjustment of *P* values by permutation-based *minP*

^a^Odds ratios were estimated by logistic regression conditioned on center of recruitment, age, menopausal status at the time of blood collection, phase of the menstrual cycle at blood collection (for premenopausal women only), use of exogenous hormone at blood collection, time of the day at blood collection, and fasting status at blood collection

^b^Multiple testing controlled for family-wise error rate at *α* = 0.05 by permutation-based stepdown minP adjustment of *P* values

^c^Multiple testing controlled for family-wise error rate at *α* = 0.05 by Bonferroni adjustment of *P* values

^d^Multiple testing controlled for false discovery rate at *α* = 0.05

When C2 and PC ae C36:3 were further analyzed as categorical variables, results similar to those of the linear analysis were obtained; logistic regression conditioned on the matching variables showed a linear trend across quintiles of C2 (OR quintile 5 versus quintile 1 = 1.54, 95% CI = 1.21–1.95, *P* trend = 0.0002) and of PC ae C36:3 (OR quintile 5 versus quintile 1 = 0.73, 95% CI = 0.58–0.91, *P* trend = 0.0003) (Table 3). Adjusting for anthropometric variables in separate models had little effect on the risk estimates (Table 3).

**Table 3:** Associations between C2 and PC ae C 36:3 and risk of breast cancer

	Cases/controls	Model 1^c^	Model 2—adjusted for WC	Model 3—adjusted for weight	Model 4—adjusted for HC
		OR (95% CI)	OR (95% CI)	OR (95% CI)	OR (95% CI)
C2					
Per SD increment	1624/1624	1.15 (1.06–1.24)	1.14 (1.06–1.23)	1.15 (1.06–1.24)	1.14 (1.06–1.24)
C2 (quintiles)^a^					
1	287/322	1.00 (ref.)	1.00 (ref.)	1.00 (ref.)	1.00 (ref.)
2	291/326	1.02 (0.81–1.28)	1.00 (0.79–1.26)	1.00 (0.80–1.27)	1.00 (0.80–1.26)
3	322/324	1.15 (0.91–1.44)	1.12 (0.89–1.41)	1.13 (0.90–1.42)	1.13 (0.89–1.42)
4	311/326	1.12 (0.89–1.41)	1.09 (0.87–1.37)	1.09 (0.87–1.37)	1.10 (0.87–1.38)
5	413/326	1.54 (1.21–1.95)	1.51 (1.19–1.91)	1.53 (1.20–1.93)	1.52 (1.20–1.93)
*P* trend^b^		0.0002	0.0005	0.0004	0.0004
PC ae C36:3					
Per SD increment	1624/1624	0.88 (0.82–0.95)	0.90 (0.83–0.97)	0.90 (0.83–0.96)	0.89 (0.83–0.96)
PC ae C36:3 (quintiles)^a^					
1	367/325	1.00 (ref.)	1.00 (ref.)	1.00 (ref.)	1.00 (ref.)
2	363/323	0.99 (0.80–1.23)	1.01 (0.82–1.25)	1.02 (0.82–1.26)	1.02 (0.82–1.26)
3	357/326	0.96 (0.77–1.19)	0.98 (0.79–1.22)	0.98 (0.79–1.22)	0.97 (0.78–1.21)
4	264/326	0.70 (0.56–0.88)	0.73 (0.58–0.91)	0.73 (0.58–0.91)	0.72 (0.58–0.91)
5	273/324	0.73 (0.58–0.91)	0.77 (0.61–0.96)	0.76 (0.61–0.96)	0.75 (0.60–0.95)
*P* trend^b^		0.0003	0.0020	0.0016	0.0010

*CI* confidence interval, *HC* hip circumference, *OR* odds ratio, *SD* standard deviation, *WC* waist circumference

^a^Quintile cut-points were determined on control participants

For log-transformed C2, cut-points were as follows, in log(μmol/L): < 1.18/1.18–1.37/1.37–1.55/1.55–1.77/≥ 1.77. For log-transformed PC ae C36:3, cut-points were as follows, in log(μmol/L): < 1.81/1.81–1.94/1.94–2.04/2.04–2.16/≥ 2.16

^b^For test of linear trends across quintiles, participants were assigned the median value in each category and the corresponding variable was modeled as a continuous term

^c^Conditional logistic regression conditioned on matching factors

### Stratification by hormone therapy

**Fig. 2:**
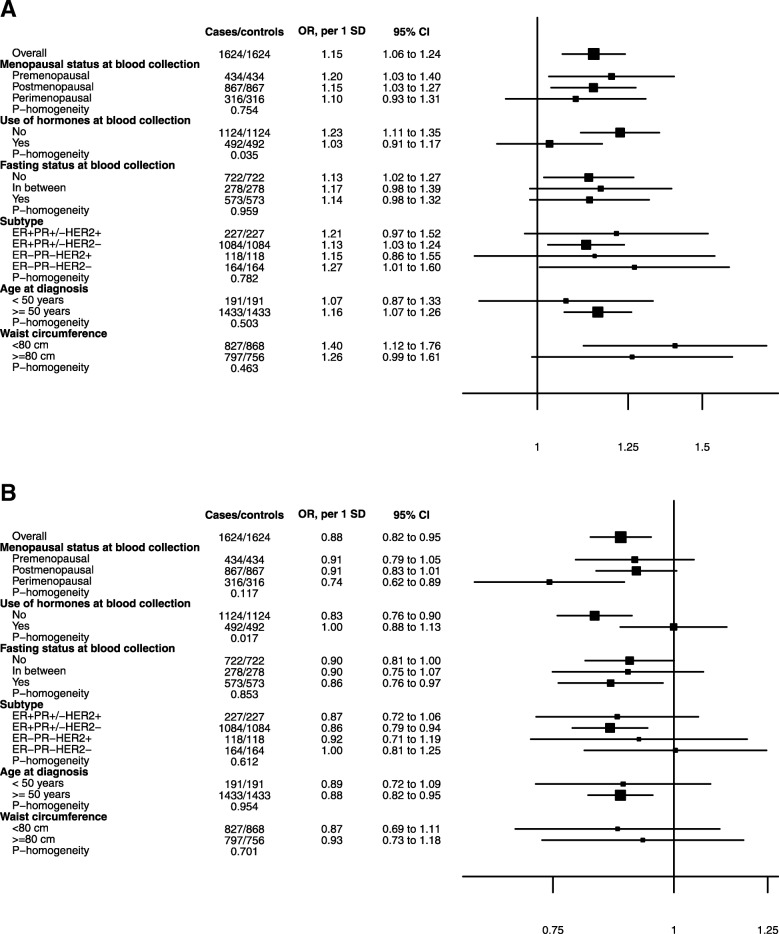
Associations between C2 (**a**) and PC ae C36:3 (**b**) and breast cancer, by selected variables. CI: confidence interval; ER: estrogen receptor; HER2: human epidermal growth factor receptor 2; PC: phosphatidylcholine; PR: progesterone receptor; SM: sphingomyelin. Odds ratios (ORs) are estimated per standard deviation (SD) increase in log-transformed metabolite concentrations, from logistic regression conditioned on matching variables. Homogeneity was tested by adding an interaction term in the conditional logistic regression model for menopausal status, use of hormones at blood collection, fasting status, breast cancer subtype, and age at diagnosis (all matching factors or case characteristics). For waist circumference (non-matching factor), logistic regression adjusted for each matching factor was used

Statistically significant heterogeneity was observed by use of hormones at blood collection for the associations of C2 (*P* homogeneity = 0.035) and PC ae C36:3 (*P* homogeneity = 0.017) with breast cancer, with statistically significant associations restricted to hormone non-users (C2: OR per SD = 1.23, 95% CI = 1.11–1.35; PC ae C36:3: OR per SD = 0.83, 95% CI = 0.76–0.90) and no associations observed in users (C2: OR per SD = 1.03, 95% CI = 0.91–1.17; PC ae C36:3: OR per SD = 1.00, 95% CI = 0.88–1.13; Fig. 2).

**Fig. 3:**
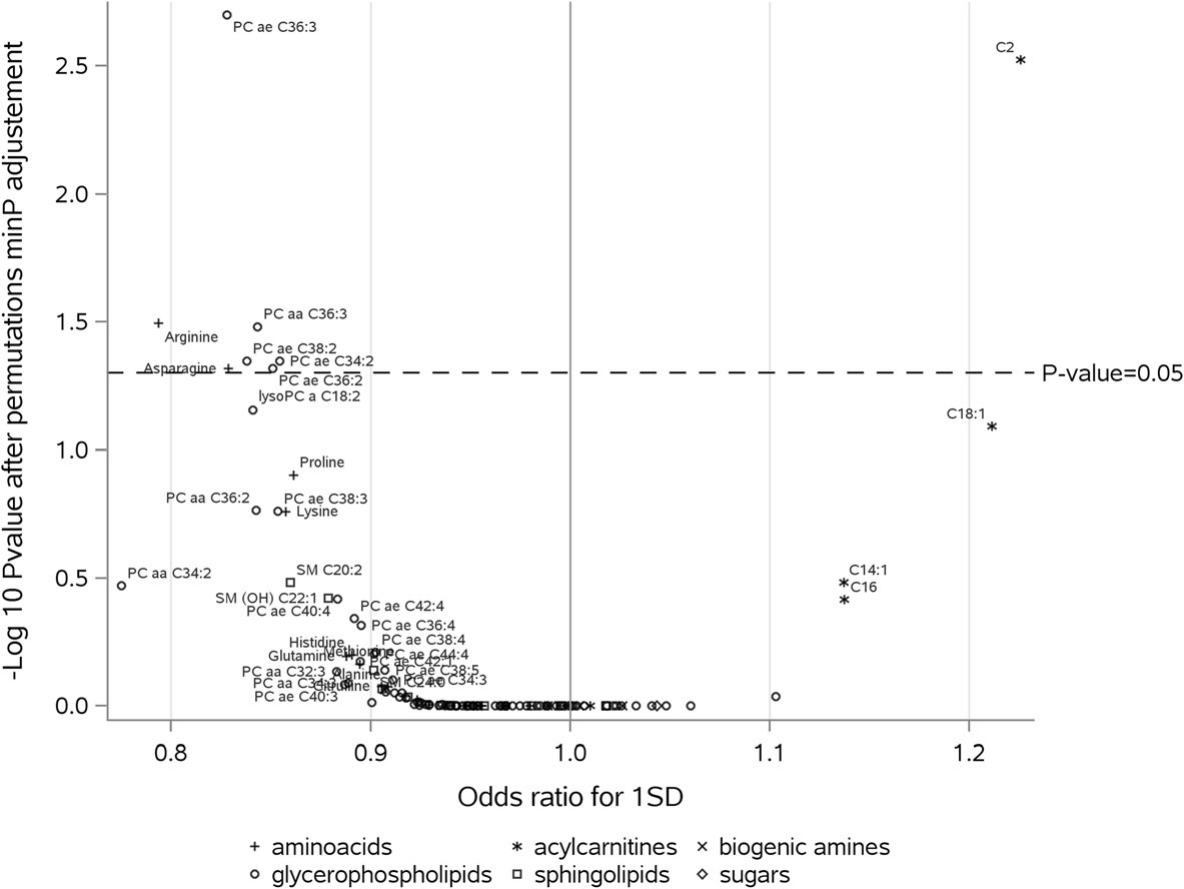
Adjusted *P* values for associations between metabolites and breast cancer, hormone non-users (1124 cases, 1124 controls). PC: phosphatidylcholine; SM: sphingomyelin. Odds ratios (ORs) are estimated per standard deviation (SD) increase in log-transformed metabolite concentrations, from logistic regression conditioned on matching variables. Raw *P* values were adjusted by permutation-based stepdown minP (see “Methods” section for details); adjusted *P* values above 0.05 (dotted line) were considered statistically significant after correction for multiple tests

In an analysis of the 127 metabolites restricted to hormone non-users (*n* = 2248) (Fig. 3), we identified additional metabolites showing statistically significant inverse associations with risk of breast cancer after adjustment of *P* values for multiple testing, for which heterogeneity was also investigated. These metabolites were as follows: arginine (OR per SD = 0.79, 95% CI = 0.70–0.90; *P* homogeneity = 0.002), asparagine (OR per SD = 0.83, 95% CI = 0.74–0.92; *P* homogeneity = 0.12), PC aa C36:3 (OR per SD = 0.84, 95% CI = 0.77–0.93; *P* homogeneity = 0.12), PC ae C34:2 (OR per SD = 0.85, 95% CI = 0.78–0.94; *P* homogeneity = 0.04), PC ae C36:2 (OR per SD = 0.85, 95% CI = 0.78–0.88; *P* homogeneity = 0.04), and PC ae C38:2 (OR per SD = 0.84, 95% CI = 0.0.76–0.93; *P* homogeneity = 0.10).

No significant heterogeneity was observed for the association of C2 and PC ae C36:3 with breast cancer by menopausal status, fasting status at blood collection, breast cancer subtype, age at diagnosis, WC (*P* homogeneity all > 0.12, Fig. 2), country (*P* homogeneity of 0.50 for C2 and 0.12 for PC ae C36:3) or by time between blood collection and diagnosis (2–8.6/≥8.6 years (median); *P* homogeneity of 0.17 for C2 and 0.98 for PC ae C36:3) (data not shown).

Stratification by breast cancer subtypes for all metabolites (see Additional file 1: Figure S2) showed that no metabolite reached statistical significance after correction for multiple testing in each subtype, although for ER+PR+/−HER2− cases (*n* = 1084 cases), PC ae C36:3 and PC aa C36:3 had adjusted *P* values close to statistical significance (0.066 and 0.074, respectively).

## Discussion

In this prospective analysis that investigated the association of 127 circulating metabolites with breast cancer incidence, among women not using hormones at baseline, and after control for multiple tests, acylcarnitine C2 was positively associated with risk of breast cancer, while levels of a set of phosphatidylcholines (ae C36:3, aa C36:3, ae C34:2, ae C36:2 and ae C38:2) and the amino acids arginine and asparagine were inversely associated with disease risk. In the overall population (hormone users and non-users), only C2 and PC ae C36:3 were associated with risk of breast cancer independently from breast cancer subtype, age at diagnosis, fasting and menopausal status at collection, or adiposity.

Acylcarnitine C2 plays a key role in the transport of fatty acids into the mitochondria for β-oxidation [27, 28]. In human intervention studies, plasma concentration levels have been seen to vary according to the activity of the fatty oxidation pathway [28, 29]. High C2 levels are associated to other known mechanisms involved in breast cancer development, such as hyperinsulinemia and insulin resistance [30], consistent with some studies showing increased plasma concentrations of acetylcarnitine in pre-diabetic or diabetic women [31–33]. An explanation for the associations observed only in women not using hormones, for C2 and for other metabolites, could be that due to their increased exposure to estrogens, MHT users are already at a higher risk of breast cancer than non-users [34], similarly to what is observed for BMI and postmenopausal breast cancer risk [35].

Phospholipids are a major component of cell membranes and play a major role in cell signaling and cell cycle regulation. Previous studies of phospholipids showed that PC ae C36:3 concentrations were decreased in type 2 diabetes [36, 37] and that lower serum levels were predictive of future diabetes [38]. Lower concentrations of PCs ae C38:2 and ae C34:2 were also observed in diabetic men compared to non-diabetics [37]. A biological basis for such inverse associations could rely on observed antioxidant effect of PCs [39].

In line with the inverse association observed between arginine and risk of breast cancer in hormone non-users, decreased plasma concentrations of arginine has been observed in breast cancer patients [40] compared with controls. Both human [41] and animal [42] studies have observed a reduction in anti-tumor immune responses in the context of arginine depletion in breast cancer, suggesting a link between arginine and immunity. In addition, higher plasma concentrations of arginine were correlated with lower estradiol and insulin-like growth factor 1 concentrations in premenopausal women [43], linking arginine to known mechanisms leading to breast cancer development. Regarding asparagine, a recent animal and in vitro study suggested that reduced asparagine bioavailability resulted in slower disease progression [44]. However, the role of asparagine in cancer development is not clear.

Prospective data on metabolomics and risk of breast cancer are limited [14, 16–18], and differences in approaches (targeted or untargeted metabolomics), analytical methods (NMR or MS), and samples (serum or plasma) make comparisons of the results difficult. Only one previous analysis used a similar targeted metabolomics approach with measurement of the same metabolites [14] and showed that lysophosphatidylcholine a C18:0 was inversely associated with risk of breast cancer after Bonferroni correction of *P* values, and that an inverse association close to statistical significance was observed for PC ae C38:1. However, none of the metabolites identified in the present work were associated with risk of breast cancer in this previous study, which did not investigate heterogeneity by use of hormones.

In a previous study applying NMR-based metabolomics analyses in the SU.VI.MAX cohort [16], several amino acids, lipoproteins, lipids, and glycerol-derived compounds were identified as significantly associated to breast cancer risk, suggesting that modifications in amino acid metabolism and energetic homeostasis in the context of setting up of insulin resistance could play a role in the disease. Results from the Prostate, Lung, Colorectal, and Ovarian Cancer Screening (PLCO) study, based on an MS-based metabolomics approach in serum samples, indicated that some metabolites correlated with alcohol intake (androgen pathway metabolites, vitamin E, and animal fats) [18], and with BMI (metabolites involved in steroid hormones metabolism and branched-chain amino acids) [17], were also associated with breast cancer risk.

Heterogeneity by subtype was investigated only in the PLCO study, showing that some metabolites (allo-isoleucine, 2-methylbutyrylcarnitine [17], etiocholanolone glucuronide, 2-hydroxy-3-mthylvalerate, pyroglutamine, 5α-androstan-3β, 17β-diol disulfate [18]) were associated with risk of ER+ breast cancer, but not with breast cancer overall, indicating that the etiology of breast cancer differs by subtype. In our work, however, we did not observe any heterogeneity of results according to receptor status of the cancers.

This study is the largest prospective investigation of metabolomics and risk of breast cancer to date. Strengths of this work include its large sample size, which allowed us to examine associations by breast cancer subtype. In addition, the exclusion of cases diagnosed less than 2 years after blood collection reduces the risk of reverse causation in our findings. Finally, the assessment of numerous lifestyle factors and anthropometric measures allowed us to examine and control for potential confounding.

A potential limitation to our work is that blood was collected from participants at one time point only. Nevertheless, the reliability of plasma metabolites analyzed here has been shown to be relatively stable over 4 months to 2 years, leading to the conclusion that a single measurement might be sufficient [45, 46, 47]. In addition, although fasting samples might be preferable over non-fasting samples, in our study, cases and controls were matched on fasting status and the results did not differ by fasting state. Another limitation is that the technologies that were used for some of the metabolites (such as PCs and lysoPCs) do not allow for a precise identification of the compounds measured, since the signal observed is not specific and may correspond to several compounds. Lastly, it is important to note that the aim of the present work was to screen metabolites associated with risk, but that further work is needed to identify the factors that influence biological levels of the metabolites associated with risk and to understand their biological connection with breast cancer development. Future studies should also integrate other molecular markers known to be linked to breast cancer to gain insight into biological mechanisms.

## Conclusions

We observed a positive association between acetylcarnitine (C2) and risk of breast cancer, and an inverse association between PC ae C36:3 and risk of breast cancer. These associations were limited to women not using hormones, as were inverse associations with arginine, asparagine, PCs aa C36:3, ae C34:2, ae C36:2, and ae C38:2. These metabolites might be biomarkers of future breast cancer development. These results need to be replicated in other epidemiological studies, and more research is needed to identify determinants of these metabolites.

## Additional files

Additional file 1: Supplementary tables describing the completeness of the metabolites measures (**Table S1.**) and metabolites concentrations by case-control status (**Table S2.**.); Supplementary figures showing age-adjusted correlations between metabolites in control participants (**Figure S1.**) and adjusted *P* values for associations between metabolites and different breast cancer subtypes (**Figure S2.**). Abbreviations: BMI: Body Mass Index; EPIC: European Prospective Investigation into Cancer and nutrition; ER: estrogen receptor; FDR: False Discovery Rate; HER2: Human epidermal growth factor receptor 2; IARC: International Agency for Research on Cancer; MHT: menopause hormone therapy; MS: Mass spectrometry; NMR: nuclear magnetic resonance; OR: odds ratio; PC: phosphatidylcholine; PR: progesterone receptor; SD: standard deviation; WC: waist circumference. (PDF 1041 kb)

## Data Availability

EPIC data are available for investigators who seek to answer important questions on health and disease in the context of research projects that are consistent with the legal and ethical standard practices of IARC/WHO and the EPIC Centres. The primary responsibility for accessing the data belongs to the EPIC Centres that provided them. For information on how to submit an application for gaining access to EPIC data and/or biospecimens, please follow the instructions at http://epic.iarc.fr/access/index.php.
