# Supplementary material for: Prospective analysis of circulating metabolites and breast cancer in EPIC
Source: BMC Med. 2019 Sep 24;17:178. doi: 10.1186/s12916-019-1408-4 (PMC6757362; doi:10.1186/s12916-019-1408-4)

Supplementary Table 1. Completeness and exclusions of the different metabolites, with limits of detection (LOD) and limits of quantification (LOQ)

Metabolites[1]	Non-quantifiable results[2]			CV[4], %
	Missing[3] data, %	Results outside the measurable range		
		Samples, %	Criteria	
**Acylcarnitines**				
C0	0	0	<LOD	4.0
C2	0	0	<LOD	4.1
C3	0	0	<LOD	5.0
C3-DC (C4-OH)	0	39.04	<LOD	-
C4	0	0.06	<LOD	5.4
C5	0	0.77	<LOD	5.4
C5-DC (C6-OH)	0	90.21	<LOD	-
C8	0	92.43	<LOD	-
C10	0	52.99	<LOD	-
C10:1	0	93.1	<LOD	-
C12	0	85.01	<LOD	-
C12:1	0	56.34	<LOD	-
C14:1	0	6.13	<LOD	9.2
C14:2	0	63.02	<LOD	-
C16	0	0.15	<LOD	5.4
C16:1	0	90.73	<LOD	-
C18	0	23.52	<LOD	-
C18:1	0	0.28	<LOD	4.8
C18:2	0	22.11	<LOD	-
**Amino acids**				
Alanine	0	0	<LOQ (< 20 µmol/L)	4.2
Arginine	0	0	<LOQ (< 5 µmol/L)	5.4
Asparagine	0	0	<LOQ (< 5 µmol/L)	4.0
Aspartate	0	34.33	<LOQ (< 5 µmol/L)	-
Citrulline	0	0	<LOQ (< 5 µmol/L)	5.9
Glutamine	0	0	<LOQ (< 20 µmol/L)	6.3
Glutamate	0	0.22	<LOQ (< 10 µmol/L)	4.7
Glycine	0	0	<LOQ (< 25 µmol/L)	6.2
Histidine	0	0	<LOQ (< 5 µmol/L)	5.7
Isoleucine	0	0	<LOQ (< 5 µmol/L)	6.8
Leucine	0	0.03	<LOQ (< 50 µmol/L)	6.2
Lysine	0	0	<LOQ (< 10 µmol/L)	8.5
Methionine	0	0	<LOQ (< 5 µmol/L)	8.4
Ornithine	0	0	<LOQ (< 5 µmol/L)	8.2
Phenylalanine	0	0	<LOQ (< 5 µmol/L)	6.0
Proline	0	0	<LOQ (< 10 µmol/L)	5.5
Serine	0	0	<LOQ (< 5 µmol/L)	5.6
Threonine	0	0	<LOQ (< 5 µmol/L)	4.2
Tryptophane	0	0	<LOQ (< 5 µmol/L)	6.2
Tyrosine	0	0	<LOQ (< 5 µmol/L)	5.6
Valine	0	0	<LOQ (< 10 µmol/L)	5.5
**Biogenic amines**				
ADMA	0	2.19	<LOQ (< 0.25 µmol/L)	8.3
Creatinine	0	0	<LOQ (< 10 µmol/L)	3.2
Kynurenine	0	0.95	<LOQ (< 1 µmol/L)	6.5
Nitro-Tyr	0	98.83	<LOQ (< 1 µmol/L)	-
Putrescine	0	72.78	<LOQ (< 0.1 µmol/L)	-
SDMA	0	0	<LOQ (< 0.1 µmol/L)	7.7
Sarcosine	0	29.65	<LOQ (< 1 µmol/L)	-
Serotonin	0	76.29	<LOQ (< 0.1 µmol/L)	-
Spermidine	0	91.72	<LOQ (< 0.25 µmol/L)	-
Spermine	0	95.72	<LOQ (< 0.25 µmol/L)	-

Metabolites[1]	Non-quantifiable results[2]			CV[4], %
	Missing[3] data, %	Results outside the measurable range		
		Samples, %	Criteria	
Taurine	0	0	<LOQ (< 2.5 µmol/L)	3.1
alpha-AAA	0	80.23	<LOQ (< 1 µmol/L)	-
t4-OH-Pro	0	0	<LOQ (< 2 µmol/L)	5.0
**Glycerophospholipids**				
PC aa C28:1	0	0	<LOD	4.5
PC aa C30:0	0	0	<LOD	4.7
PC aa C32:0	0	0	<LOD	5.1
PC aa C32:1	0	0	<LOD	5.5
PC aa C32:2	0	0	<LOD	6.0
PC aa C32:3	0	0	<LOD	5.5
PC aa C34:1	0	0	<LOD	5.3
PC aa C34:2	0	0	<LOD	6.3
PC aa C34:3	0	0	<LOD	5.1
PC aa C34:4	0	0	<LOD	5.4
PC aa C36:0	0	0.06	<LOD	6.6
PC aa C36:1	0	0	<LOD	4.6
PC aa C36:2	0	0	<LOD	4.5
PC aa C36:3	0	0	<LOD	4.4
PC aa C36:4	0	0	<LOD	4.4
PC aa C36:5	0	0	<LOD	4.7
PC aa C36:6	0	0	<LOD	5.3
PC aa C38:0	0	0	<LOD	4.5
PC aa C38:3	0	0	<LOD	3.9
PC aa C38:4	0	0	<LOD	4.0
PC aa C38:5	0	0	<LOD	4.1
PC aa C38:6	0	0	<LOD	4.0
PC aa C40:1	0	69.77	<LOD	-
PC aa C40:2	0	0	<LOD	7.2
PC aa C40:3	0	0	<LOD	5.6
PC aa C40:4	0	0	<LOD	4.1
PC aa C40:5	0	0	<LOD	4.3
PC aa C40:6	0	0	<LOD	4.0
PC aa C42:0	0	0	<LOD	5.3
PC aa C42:1	0	0	<LOD	5.7
PC aa C42:2	0	0.03	<LOD	4.5
PC aa C42:4	0	0	<LOD	6.7
PC aa C42:5	0	0	<LOD	5.7
PC aa C42:6	0	0	<LOD	5.3
PC ae C30:0	0	0	<LOD	5.8
PC ae C30:2	0	0	<LOD	7.9
PC ae C32:1	0	0	<LOD	5.3
PC ae C32:2	0	0	<LOD	5.5
PC ae C34:0	0	0	<LOD	5.6
PC ae C34:1	0	0	<LOD	5.1
PC ae C34:2	0	0	<LOD	5.3
PC ae C34:3	0	0	<LOD	5.1
PC ae C36:0	0	0	<LOD	6.2
PC ae C36:1	0	0	<LOD	4.7
PC ae C36:2	0	0	<LOD	4.8
PC ae C36:3	0	0	<LOD	4.7
PC ae C36:4	0	0	<LOD	5.0
PC ae C36:5	0	0	<LOD	4.9
PC ae C38:0	0	0	<LOD	4.4
PC ae C38:2	0	0	<LOD	6.4
PC ae C38:3	0	0	<LOD	4.3
PC ae C38:4	0	0	<LOD	4.3

Metabolites[1]	Non-quantifiable results[2]			CV[4], %
	Missing[3] data, %	Results outside the measurable range		
		Samples, %	Criteria	
PC ae C38:5	0	0	<LOD	5.1
PC ae C38:6	0	0	<LOD	4.7
PC ae C40:1	0	0	<LOD	5.5
PC ae C40:2	0	0	<LOD	4.2
PC ae C40:3	0	0	<LOD	4.1
PC ae C40:4	0	0	<LOD	4.4
PC ae C40:5	0	0	<LOD	4.2
PC ae C40:6	0	0	<LOD	4.3
PC ae C42:1	0	0.09	<LOD	6.0
PC ae C42:2	0	0	<LOD	5.5
PC ae C42:3	0	0	<LOD	5.7
PC ae C42:4	0	0	<LOD	5.3
PC ae C42:5	0	0	<LOD	3.6
PC ae C44:3	0	0	<LOD	9.5
PC ae C44:4	0	0.03	<LOD	7.3
PC ae C44:5	0	0	<LOD	4.8
PC ae C44:6	0	0	<LOD	4.9
lysoPC a C16:0	0	0	<LOD	6.4
lysoPC a C16:1	0	0	<LOD	6.4
lysoPC a C17:0	0	0	<LOD	6.7
lysoPC a C18:0	0	0	<LOD	6.4
lysoPC a C18:1	0	0	<LOD	6.5
lysoPC a C18:2	0	0	<LOD	6.3
lysoPC a C20:3	0	0	<LOD	6.0
lysoPC a C20:4	0	0	<LOD	5.8
lysoPC a C24:0	0	0	<LOD	13.5
lysoPC a C28:1	0	0	<LOD	11.7
**Sphingolipids**				
SM C16:0	0	0	<LOD	5.2
SM C16:1	0	0	<LOD	5.3
SM C18:0	0	0	<LOD	5.1
SM C18:1	0	0	<LOD	5.0
SM C20:2	0	0	<LOD	8.0
SM C24:0	0	0	<LOD	5.5
SM C24:1	0	0	<LOD	4.8
SM C26:0	0	0	<LOD	9.8
SM C26:1	0	0	<LOD	7.9
SM (OH) C14:1	0	0	<LOD	5.8
SM (OH) C16:1	0	0	<LOD	5.1
SM (OH) C22:1	0	0	<LOD	5.0
SM (OH) C22:2	0	0	<LOD	4.9
SM (OH) C24:1	0	0	<LOD	6.0
**Hexose**				
Hexose	0	2.03	<LOQ (< 1500 µmol/L)	3.7

[1]Metabolites in gray were excluded from the statistical analysis because more than 20% or the measures were outside the measurable range.

[2] Limit of detection (LOD) is applicable to acylcarnitines, glycerophospholipids, sphingolipids. Limit of quantification (LOQ) is applicable to amino acids, biogenic amines and hexose. No results was reported to be higher than the upper limit of quantification.

[3]Missing values were defined as unmeasured metabolite in samples because of technical problems.

[4]Mean intra- and inter-batch coefficients of variation (CVs) based on two quality control samples measured in duplicate in each batch.

Supplementary Table 2. Description of metabolites concentrations by case-control status

Metabolites	Cases<br>N=1624<br>Geometric mean (95% CI) - µmol/L	Controls<br>N=1624<br>Geometric mean (95% CI) - µmol/L
**Acylcarnitines**		
C0	28.75 (28.43-29.07)	28.65 (28.33-28.97)
C2	4.57 (4.48-4.65)	4.38 (4.30-4.46)
C3	0.27 (0.26-0.27)	0.27 (0.26-0.27)
C4	0.15 (0.14-0.15)	0.15 (0.14-0.15)
C5	0.10 (0.09-0.10)	0.10 (0.09-0.10)
C14:1	0.06 (0.06-0.06)	0.06 (0.06-0.06)
C16	0.10 (0.09-0.10)	0.09 (0.09-0.10)
C18:1	0.10 (0.10-0.11)	0.10 (0.10-0.10)
**Amino acids**		
Alanine	308.30 (304.61-312.03)	311.99 (308.20-315.82)
Arginine	54.87 (53.59-56.19)	56.16 (54.88-57.47)
Asparagine	39.11 (38.65-39.58)	39.97 (39.50-40.44)
Citrulline	26.17 (25.82-26.52)	26.59 (26.23-26.95)
Glutamine	478.89 (474.29-483.53)	485.02 (480.38-489.70)
Glutamate	45.60 (44.07-47.19)	45.80 (44.26-47.39)
Glycine	201.74 (198.65-204.88)	207.59 (204.35-210.87)
Histidine	71.95 (71.39-72.52)	72.80 (72.20-73.40)
Isoleucine	58.50 (57.67-59.35)	58.76 (57.90-59.63)
Leucine	108.31 (106.94-109.70)	108.61 (107.19-110.04)
Lysine	180.44 (178.34-182.56)	183.83 (181.74-185.94)
Methionine	19.36 (19.12-19.61)	19.54 (19.28-19.80)
Ornithine	73.26 (71.96-74.58)	74.17 (72.84-75.53)
Phenylalanine	54.72 (54.21-55.23)	54.86 (54.34-55.38)
Proline	155.81 (153.43-158.23)	158.62 (156.10-161.19)
Serine	91.38 (90.34-92.43)	92.31 (91.25-93.39)
Threonine	106.52 (105.20-107.86)	108.38 (107.05-109.73)
Tryptophane	51.50 (51.02-51.99)	51.75 (51.28-52.23)
Tyrosine	58.99 (58.22-59.78)	58.91 (58.12-59.70)
Valine	184.04 (182.12-185.97)	184.01 (182.10-185.93)
**Biogenic amines**		
ADMA	0.38 (0.38-0.38)	0.38 (0.38-0.39)
Creatinine	54.07 (53.63-54.51)	54.25 (53.78-54.73)
Kynurenine	1.89 (1.87-1.92)	1.91 (1.88-1.93)
SDMA	0.35 (0.35-0.35)	0.35 (0.35-0.35)
Taurine	49.48 (48.59-50.39)	49.42 (48.51-50.35)
t4-OH-Pro	7.76 (7.59-7.92)	7.71 (7.55-7.88)
**Glycerophospholipids**		
PC aa C28:1	2.34 (2.31-2.37)	2.34 (2.31-2.37)
PC aa C30:0	3.22 (3.16-3.27)	3.24 (3.19-3.29)
PC aa C32:0	13.48 (13.34-13.61)	13.55 (13.41-13.68)
PC aa C32:1	15.06 (14.69-15.44)	14.96 (14.58-15.34)
PC aa C32:2	4.24 (4.16-4.32)	4.33 (4.24-4.41)
PC aa C32:3	0.59 (0.58-0.59)	0.59 (0.59-0.60)
PC aa C34:1	178.36 (176.52-180.22)	178.42 (176.57-180.28)
PC aa C34:2	269.59 (266.86-272.35)	271.29 (268.59-274.01)
PC aa C34:3	15.27 (15.03-15.52)	15.52 (15.27-15.77)
PC aa C34:4	1.78 (1.75-1.81)	1.81 (1.78-1.84)
PC aa C36:0	1.86 (1.82-1.89)	1.85 (1.81-1.88)
PC aa C36:1	42.48 (41.96-43.01)	42.99 (42.46-43.52)
PC aa C36:2	189.43 (187.79-191.09)	191.91 (190.28-193.57)
PC aa C36:3	117.37 (116.26-118.49)	119.62 (118.53-120.72)
PC aa C36:4	151.52 (149.99-153.07)	151.57 (149.99-153.17)

Metabolites	Cases N=1624 Geometric mean (95% CI) - µmol/L	Controls N=1624 Geometric mean (95% CI) - µmol/L
PC aa C36:5	20.44 (19.92-20.98)	20.09 (19.58-20.61)
PC aa C36:6	0.99 (0.97-1.01)	0.99 (0.97-1.01)
PC aa C38:0	2.17 (2.14-2.20)	2.19 (2.16-2.22)
PC aa C38:3	36.80 (36.34-37.27)	37.43 (36.97-37.89)
PC aa C38:4	76.85 (75.95-77.76)	77.43 (76.54-78.33)
PC aa C38:5	40.20 (39.70-40.70)	40.34 (39.84-40.84)
PC aa C38:6	68.00 (66.97-69.06)	67.24 (66.20-68.31)
PC aa C40:2	0.20 (0.20-0.20)	0.20 (0.20-0.21)
PC aa C40:3	0.36 (0.36-0.37)	0.37 (0.36-0.37)
PC aa C40:4	2.13 (2.10-2.16)	2.16 (2.14-2.19)
PC aa C40:5	5.47 (5.40-5.55)	5.53 (5.46-5.61)
PC aa C40:6	19.53 (19.22-19.85)	19.44 (19.13-19.77)
PC aa C42:0	0.34 (0.34-0.35)	0.35 (0.34-0.35)
PC aa C42:1	0.16 (0.16-0.17)	0.17 (0.16-0.17)
PC aa C42:2	0.14 (0.14-0.14)	0.14 (0.14-0.14)
PC aa C42:4	0.10 (0.10-0.10)	0.10 (0.10-0.10)
PC aa C42:5	0.20 (0.20-0.20)	0.20 (0.20-0.20)
PC aa C42:6	0.30 (0.29-0.30)	0.30 (0.29-0.30)
PC ae C30:0	0.27 (0.27-0.28)	0.28 (0.27-0.28)
PC ae C30:2	0.08 (0.08-0.08)	0.08 (0.08-0.08)
PC ae C32:1	2.61 (2.58-2.64)	2.60 (2.57-2.63)
PC ae C32:2	0.75 (0.74-0.76)	0.75 (0.74-0.76)
PC ae C34:0	1.51 (1.49-1.53)	1.53 (1.51-1.55)
PC ae C34:1	9.61 (9.51-9.71)	9.67 (9.57-9.77)
PC ae C34:2	11.32 (11.20-11.45)	11.56 (11.43-11.70)
PC ae C34:3	7.50 (7.40-7.60)	7.62 (7.52-7.72)
PC ae C36:0	0.73 (0.72-0.74)	0.73 (0.73-0.74)
PC ae C36:1	7.82 (7.72-7.92)	7.87 (7.77-7.97)
PC ae C36:2	14.01 (13.85-14.17)	14.29 (14.13-14.45)
PC ae C36:3	7.12 (7.05-7.19)	7.30 (7.22-7.37)
PC ae C36:4	14.00 (13.85-14.15)	14.20 (14.04-14.35)
PC ae C36:5	9.42 (9.31-9.53)	9.43 (9.32-9.54)
PC ae C38:0	1.82 (1.79-1.84)	1.82 (1.79-1.85)
PC ae C38:2	1.68 (1.66-1.71)	1.73 (1.70-1.75)
PC ae C38:3	3.73 (3.68-3.78)	3.80 (3.75-3.85)
PC ae C38:4	10.31 (10.21-10.41)	10.43 (10.34-10.54)
PC ae C38:5	12.80 (12.67-12.93)	12.97 (12.84-13.10)
PC ae C38:6	6.00 (5.93-6.07)	6.03 (5.95-6.10)
PC ae C40:1	0.90 (0.89-0.91)	0.91 (0.90-0.93)
PC ae C40:2	1.37 (1.35-1.39)	1.37 (1.36-1.39)
PC ae C40:3	0.79 (0.78-0.80)	0.80 (0.79-0.81)
PC ae C40:4	1.59 (1.57-1.61)	1.62 (1.60-1.63)
PC ae C40:5	2.55 (2.52-2.59)	2.57 (2.54-2.60)
PC ae C40:6	3.56 (3.52-3.61)	3.59 (3.54-3.63)
PC ae C42:1	0.23 (0.23-0.23)	0.23 (0.23-0.24)
PC ae C42:2	0.35 (0.34-0.35)	0.35 (0.35-0.36)
PC ae C42:3	0.51 (0.50-0.52)	0.52 (0.51-0.52)
PC ae C42:4	0.49 (0.49-0.50)	0.50 (0.49-0.50)
PC ae C42:5	1.31 (1.30-1.33)	1.32 (1.31-1.33)
PC ae C44:3	0.08 (0.08-0.08)	0.08 (0.08-0.08)
PC ae C44:4	0.20 (0.20-0.20)	0.20 (0.20-0.20)
PC ae C44:5	0.81 (0.80-0.82)	0.80 (0.79-0.81)
PC ae C44:6	0.62 (0.61-0.63)	0.62 (0.61-0.63)
lysoPC a C16:0	76.32 (75.34-77.32)	76.90 (75.92-77.89)
lysoPC a C16:1	2.28 (2.25-2.32)	2.29 (2.25-2.32)

Metabolites	Cases	Controls
	N=1624	N=1624
	Geometric mean (95% CI) - µmol/L	Geometric mean (95% CI) - µmol/L
lysoPC a C17:0	1.55 (1.52-1.57)	1.57 (1.54-1.60)
lysoPC a C18:0	22.89 (22.52-23.27)	23.35 (22.97-23.74)
lysoPC a C18:1	15.67 (15.42-15.91)	15.88 (15.63-16.13)
lysoPC a C18:2	21.75 (21.37-22.14)	22.41 (22.02-22.80)
lysoPC a C20:3	1.64 (1.61-1.66)	1.67 (1.65-1.70)
lysoPC a C20:4	4.30 (4.24-4.36)	4.32 (4.25-4.38)
lysoPC a C24:0	0.11 (0.11-0.11)	0.11 (0.11-0.11)
lysoPC a C28:1	0.31 (0.30-0.31)	0.31 (0.31-0.32)
**Sphingolipids**		
SM C16:0	84.24 (83.56-84.93)	84.72 (84.03-85.41)
SM C16:1	12.30 (12.18-12.42)	12.33 (12.21-12.46)
SM C18:0	17.01 (16.83-17.20)	17.08 (16.89-17.26)
SM C18:1	8.54 (8.44-8.63)	8.58 (8.49-8.68)
SM C20:2	0.39 (0.39-0.40)	0.41 (0.40-0.42)
SM C24:0	11.22 (11.10-11.34)	11.37 (11.25-11.49)
SM C24:1	25.41 (25.14-25.67)	25.31 (25.05-25.57)
SM C26:0	0.09 (0.08-0.09)	0.09 (0.09-0.09)
SM C26:1	0.18 (0.18-0.19)	0.18 (0.18-0.19)
SM (OH) C14:1	5.03 (4.97-5.09)	5.04 (4.98-5.11)
SM (OH) C16:1	2.73 (2.70-2.77)	2.75 (2.71-2.78)
SM (OH) C22:1	8.58 (8.49-8.68)	8.74 (8.65-8.84)
SM (OH) C22:2	7.07 (6.99-7.14)	7.15 (7.07-7.22)
SM (OH) C24:1	0.71 (0.70-0.72)	0.72 (0.71-0.73)
**Sugars**		
Hexose	3978.9 (3918.2-4040.6)	3915.1 (3852.0-3979.2)

Abbreviations: CI, confidence interval; PC, phosphatidylcholine; SM, sphingomyelin.

Supplementary Figure 1. Age-adjusted partial correlations between metabolites among controls (N=1624)

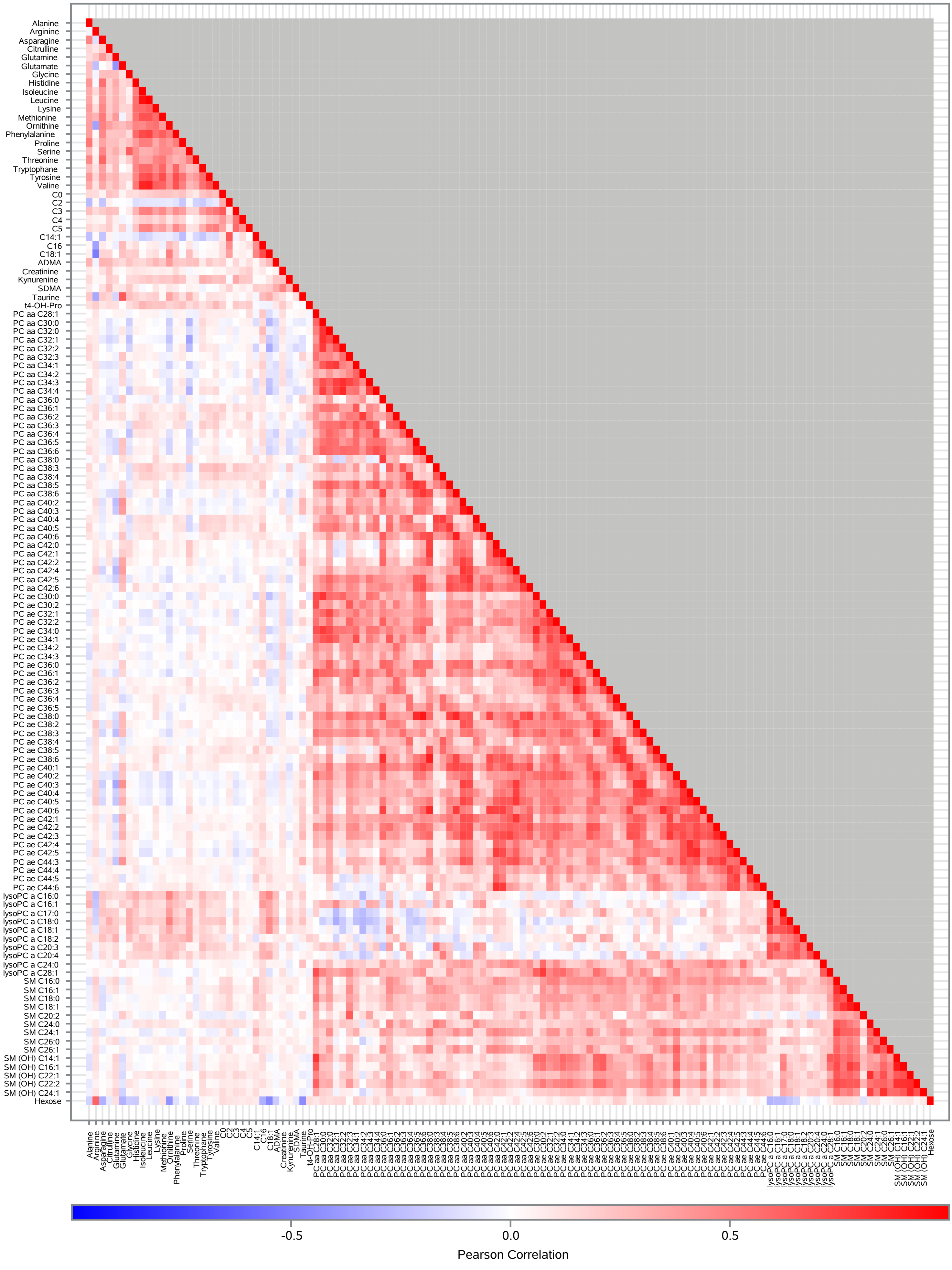

Supplementary Figure 2. Permutation-based stepdown minP adjusted P-values for association between metabolites and risk of breast cancer, by subtype

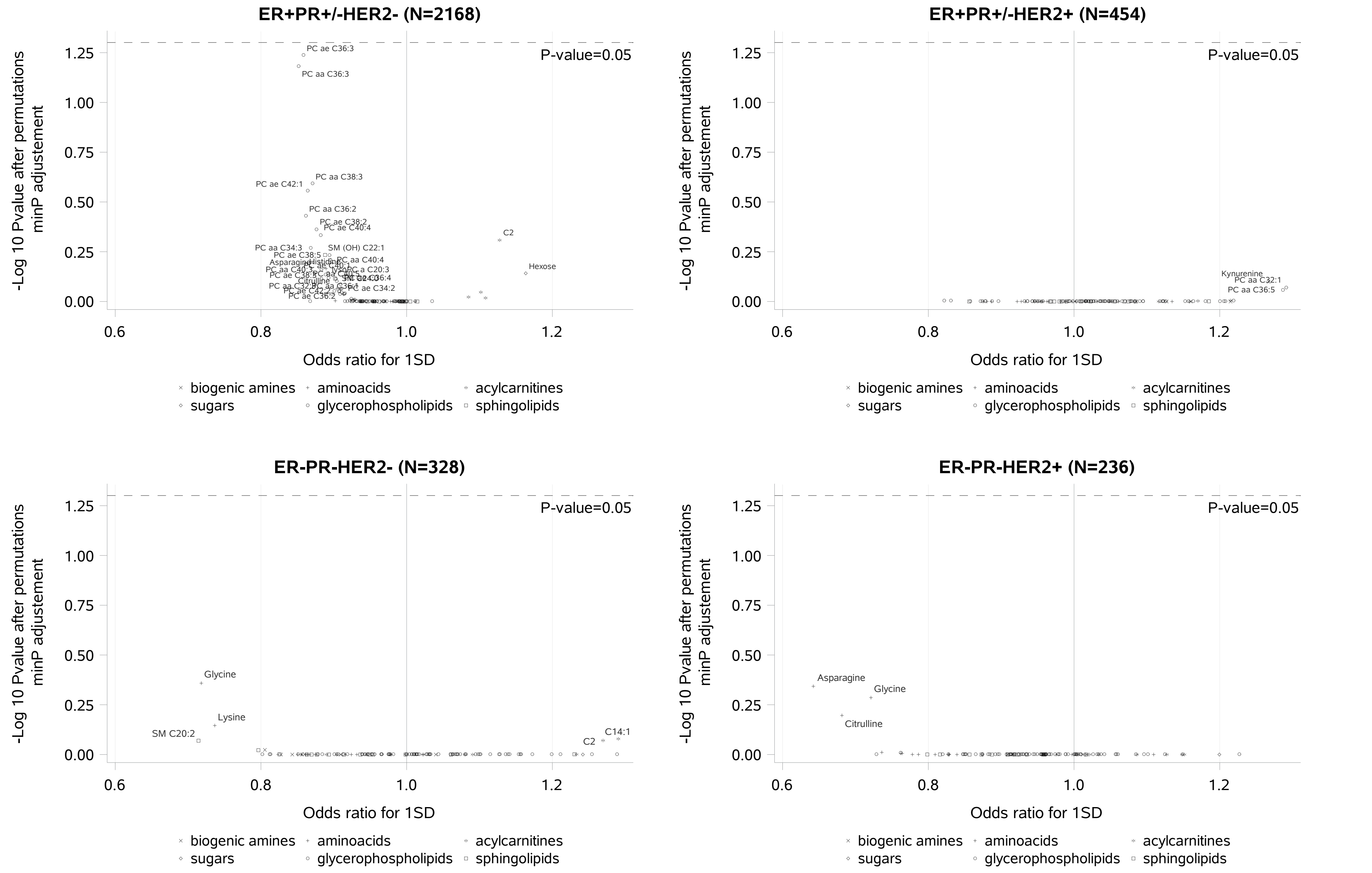

Supplement: Supplementary file 1 — Supplementary tables describing the completeness of the metabolites measures (Table S1.) and metabolites concentrations by case-control status (Table S2..); Supplementary figures showing age-adjusted correlations between metabolites in control participants (Figure S1.) and adjusted P values for associations between metabolites and different breast cancer subtypes (Figure S2.). Abbreviations: BMI: Body Mass Index; EPIC: European Prospective Investigation into Cancer and nutrition; ER: estrogen receptor; FDR: False Discovery Rate; HER2: Human epidermal growth factor receptor 2; IARC: International Agency for Research on Cancer; MHT: menopause hormone therapy; MS: Mass spectrometry; NMR: nuclear magnetic resonance; OR: odds ratio; PC: phosphatidylcholine; PR: progesterone receptor; SD: standard deviation; WC: waist circumference. (PDF 1041 kb) [file 12916_2019_1408_MOESM1_ESM.pdf]
